# Establishing an adjusted p-value threshold to control the family-wide type 1 error in genome wide association studies

**DOI:** 10.1186/1471-2164-9-516

**Published:** 2008-10-31

**Authors:** Priya Duggal, Elizabeth M Gillanders, Taura N Holmes, Joan E Bailey-Wilson

**Affiliations:** 1 Statistical Genetics Section, Inherited Disease Research Branch, National Human Genome Research Institute, National Institutes of Health, Baltimore, MD USA

## Abstract

**Background:**

By assaying hundreds of thousands of single nucleotide polymorphisms, genome wide association studies (GWAS) allow for a powerful, unbiased review of the entire genome to localize common genetic variants that influence health and disease. Although it is widely recognized that some correction for multiple testing is necessary, in order to control the family-wide Type 1 Error in genetic association studies, it is not clear which method to utilize. One simple approach is to perform a Bonferroni correction using all *n single nucleotide polymorphisms (*SNPs) across the genome; however this approach is highly conservative and would "overcorrect" for SNPs that are not truly independent. Many SNPs fall within regions of strong linkage disequilibrium (LD) ("blocks") and should not be considered "independent".

**Results:**

We proposed to approximate the number of "independent" SNPs by counting 1 SNP per LD block, plus all SNPs outside of blocks (interblock SNPs). We examined the *effective *number of independent SNPs for Genome Wide Association Study (GWAS) panels. In the CEPH Utah (CEU) population, by considering the interdependence of SNPs, we could reduce the total number of effective tests within the Affymetrix and Illumina SNP panels from 500,000 and 317,000 to 67,000 and 82,000 "independent" SNPs, respectively. For the Affymetrix 500 K and Illumina 317 K GWAS SNP panels we recommend using 10^-5^, 10^-7 ^and 10^-8 ^and for the Phase II HapMap CEPH Utah and Yoruba populations we recommend using 10^-6^, 10^-7 ^and 10^-9 ^as "suggestive", "significant" and "highly significant" p-value thresholds to properly control the family-wide Type 1 error.

**Conclusion:**

By approximating the effective number of independent SNPs across the genome we are able to 'correct' for a more accurate number of tests and therefore develop 'LD adjusted' Bonferroni corrected p-value thresholds that account for the interdepdendence of SNPs on well-utilized commercially available SNP "chips". These thresholds will serve as guides to researchers trying to decide which regions of the genome should be studied further.

## Background

Since first proposed in 1996 by Risch and Merikangas [1], it has increasingly been accepted that association studies are powerful to detect modest effects of common alleles involved in complex trait susceptibility. Until recently, genotype-phenotype tests of association have been limited to candidate genes. Recent advances in molecular technologies and the availability of the human genome sequence have revolutionized researchers' ability to catalogue human genetic variation. In addition, the International HapMap project has provided researchers with invaluable information regarding the linkage disequilibrium (LD) structure within the genome [2,3]. These advances have made genome wide association studies (GWAS) to identify common variants a reality. However many issues regarding the design, analysis and interpretation of results remain to be investigated.

In particular, interpretation of results is not trivial in light of the scale of multiple testing proposed. Testing such a large number of SNPs will require a balance between power and the chance of making false discoveries. There are many methods that have been proposed to address the multiple testing issue. These include false discovery rate (FDR), permutation testing, Bayesian factors (BF) and the Bonferonni correction. The FDR controls the expected proportion of false positives among all rejections, providing a less stringent control of the Type I error [4]. The application of the FDR method specifically in the context of genome wide studies has been proposed [4-6]. Permutation testing, in which the datasets are permuted thousands of times to achieve genomewide significance is another method that has been used in candidate gene studies and now genome wide association studies [7,8]. Although empirical p-values have a theoretical advantage they may be computationally infeasible with large datasets. Another proposed method is the use of Bayesian Factors (BF) instead of frequentist p-values which need to be interpreted with the power of the study. However, BF also requires an assumption about the effect size, but the major advantage is that it can be compared across studies [9]. A simple method to control the family-wise error rate is the Bonferroni correction, which adjusts the Type 1 error (a) by the total number of tests (a/n). The Bonferroni correction can use the actual number of tests performed (i.e. SNPs genotyped) or a theoretical value based on the total number of tests possible (i.e. all SNPs). One critical, but often overlooked, assumption, of the Bonferroni correction method, is the assumption that all the tests are independent [10]. Biologically, we know that SNPs in close proximity are not independent, and therefore we are "overcorrecting" when we use the traditional Bonferroni method to adjust significance thresholds for multiple testing in GWAS studies [11]. We propose Bonferroni corrected p-value thresholds that account for the interdepdendence of SNPs on commonly used commercially available SNP "chips" (Illumina 317 K and Affymetrix 500 K) and in the HapMap panels. This method is an extension of the Bonferroni correction that accounts for the underlying linkage disequilibrium or dependence in dense SNP panels. These thresholds will be invaluable to researchers as they can be used as a guide to identifying regions of interest or significance in genome wide association studies, which should be studied further.

## Methods

In order to estimate the effective number of "independent" SNPs in 3 autosomal marker panels (HapMap, Illumina 317 K and Affymetrix 500 K) we downloaded genotype data from release 22 of the International HapMap project. We used the non-redundant CEU and YRI data mapped against the "rs strand" of build 36 of the human genome. For the Illumina and Affymetrix marker sets we used a perl script to generate chromosome specific files containing only the subset of specific markers included in the Illumina 317 K or Affymetrix 500 K panels using CEU data. Then for each chromosome of data we used a perl script to generate smaller more manageable files each containing genotype data for approximately 2500 SNPs. We used Haploview version 4.0 to evaluate blocks of linkage disequilibrium (LD) using the 'Solid Spine of LD' algorithm with a minimum D' value of 0.8. The Solid Spine of LD method internal to Haploview defines a block when the first and last markers are in strong LD with all intermediate markers. We also evaluated chromosome 1 for the CEU HapMap data using the "Solid Spine of LD' algorithm and varying the minimum D' value to 0.7 and 0.9 to determine if this value altered the thresholds. In addition, we evaluated chromosome 1 for the CEU HapMap data using the Gabriel and 4-gamete block defining methods. For all analyses we ignored pairwise comparisons of markers >500 kb apart and excluded individuals with >50% missing genotypes. We also excluded markers with a minor allele frequency less than 0.01, a Hardy-Weinberg equilibrium p-value less than 0.001 or a genotype call rate less than 75%. We then summarized across the genome: Total number of SNPs, Total number of Blocks, Total number of SNPs not in a block (inter-block SNPs) and Total number of blocks + interblock SNPs for each panel. Our programs are available upon request so that thresholds can be established per population.

## Results and discussion

We established three thresholds that correspond to 1) suggestive association in which we expect 1 false positive association per GWAS 2) significant association in which we expect one false positive association to occur 0.05 times per GWAS and 3) highly significant association in which we expect one false positive association to occur 0.001 times per GWAS. In the CEPH Utah (CEU) population, by considering the interdependence of SNPs, we reduced the total number of effective tests within the Affymetrix and Illumina SNP panels from 500,000 and 317,000 to 67,000 and 82,000 "independent" SNPs, respectively (Tables 1 and 2). This results in p-value thresholds of ≈10^-5^, 10^-7 ^and 10^-8 ^for both the Affymetrix and Illumina SNP panels (Table 3) compared to ≈10^-6^, 10^-7 ^and 10^-9 ^if we do not correct for the lack of independence among SNPs. For researchers using these set genome-wide SNP panels this provides valuable thresholds to interpret association results, and to identify SNPs that may be important for replication.

**Table 1:** Affymetrix 500 K using CEU HapMap Samples

	**Affymetrix 500,000 SNP Panel (CEU)**			
**Chromosome**	**Total number of SNPs**	**Total number of blocks**	**Total number Interblock SNPS**	**Total number of blocks + Interblock SNPs**

1	31876	4447	833	5280
2	33610	4626	787	5413
3	27588	3903	723	4626
4	25811	3514	689	4203
5	26548	3601	646	4247
6	26550	3487	604	4091
7	21544	3061	618	3679
8	22550	3053	563	3616
9	19086	2664	541	3205
10	23531	3046	510	3556
11	21477	2761	528	3289
12	20549	2821	499	3320
13	15700	2116	392	2508
14	12839	1820	371	2191
15	11560	1857	396	2253
16	12339	1944	454	2398
17	8473	1385	344	1729
18	11966	1748	374	2122
19	5177	954	305	1259
20	10292	1519	331	1850
21	5873	843	204	1047
22	5053	828	213	1041

**Total**	**399,992**	**55,998**	**10,925**	**66,923**

**Table 2:** Illumina 317 K SNPs using CEU HapMap Samples

	**Illumina 317,000 SNP Panel (CEU)**			
**Chromosome**	**Total number of SNPs**	**Total number of blocks**	**Total number Interblock SNPS**	**Total number of blocks + Interblock SNPs**

1	23055	4959	1336	6295
2	25103	5258	1348	6606
3	21332	4505	1268	5773
4	18923	3979	1055	5034
5	19062	3966	979	4945
6	20524	4044	950	4994
7	16493	3472	977	4449
8	18053	3658	940	4598
9	15691	3305	936	4241
10	15423	3263	899	4162
11	14498	3037	827	3864
12	14844	3097	918	4015
13	11411	2373	620	2993
14	9767	2086	592	2678
15	8817	1942	631	2573
16	8924	2078	705	2783
17	8279	1859	603	2462
18	10390	2183	678	2861
19	5833	1408	545	1953
20	7758	1736	496	2232
21	5430	1130	318	1448
22	5398	1156	379	1535

**Total**	**305,008**	**64,494**	**18,000**	**82,494**

**Table 3:** Thresholds for Genome Wide Association Using CEU and YRI Population Samples

**Panel**	***Suggestive *p values (1)**	***Significant *p values (0.05)**	***Highly Significant *p values (0.001)**
Affymetrix CEU 500 K (n = 66,923)	1.49 × 10^-05^	7.47 × 10^-07^	1.49 × 10^-08^

Illumina 317 K (n = 82,494)	1.21 × 10^-05^	6.06 × 10^-07^	1.21 × 10^-08^

HapMap YRI (n = 289,175)	3.45 × 10^-06^	1.73 × 10^-07^	3.45 × 10^-09^

HapMap CEU (n = 164,296)	6.09 × 10^-06^	3.04 × 10^-07^	6.09 × 10^-09^

HapMap CEU (D' > 0.7)*	8.37 × 10^-06^	4.19 × 10^-07^	8.37 × 10^-09^

HapMap CEU (D' > 0.9)*	4.38 × 10^-06^	2.19 × 10^-07^	4.38 × 10^-09^

*extrapolated from Chromosome 1 data. P-values in parentheses in the header line indicate the family-wide error rate that corresponds to the Bonferroni-corrected significance thresholds given in the columns below.

In addition to the established SNP panels, we evaluated the number of "independent" tests within the Phase II HapMap publicly available data for both the CEPH from Utah (CEU) and Yoruba (YRI) populations. Since our proposed thresholds are LD block dependent, they are population specific and the total number of "independent" SNPs may vary across populations and therefore should be considered separately. The publicly available data includes 2.4 million (CEU) and 2.7 million (YRI) SNPs across the genome. We reduced the total number of tests to 164,000 SNPs and 289,000 SNPs for the CEU and YRI, respectively (Tables 4 and 5). This results in p-value thresholds of ≈10^-6^, 10^-7 ^and 10^-9 ^for both the CEU and YRI populations (Table 3) compared to ≈10^-7^, 10^-8 ^and 10^-10 ^if we do not correct for the lack of independence among SNPs. The total number of "independent" SNPs for the YRI population is nearly double that for the CEU, however this does not have an impact on the exponent of the p-value. As expected, as the density of SNPs increases, the average number of SNPs within a block also increases. Therefore, it is likely that the additional Affymetrix and Illumina SNP panels (1 million and 650,000 SNPs) will not greatly increase the number of independent SNPs but will increase the number of SNPs within a block. However, using the highly dense HapMap population (Tables 4 and 5) provides us with thresholds that can be used for denser platforms (e.g. 1 million SNPs) or for studies that utilize statistical methods to impute the 2.5 million+ HapMap SNPs.

**Table 4:** HapMap SNPs using CEU HapMap Samples

	**CEPH Utah HapMap Samples**			
**Chromosome**	**Total number of SNPs**	**Total number of blocks**	**Total number Interblock SNPS**	**Total number of blocks + Interblock SNPs**

1	184403	10740	1815	12555
2	211913	11219	1510	12729
3	166801	9431	1426	10857
4	155953	10204	1745	10363
5	161666	8725	1238	9963
6	174458	8677	1743	10420
7	137148	8050	1140	9190
8	141925	7707	1076	8783
9	116824	7092	1105	8197
10	132087	7428	1250	8607
11	124354	6821	1037	7858
12	118973	6959	991	7950
13	99669	5290	793	6083
14	80500	4690	893	5583
15	69104	4690	814	5504
16	68205	5212	817	6029
17	56026	4127	715	4842
18	73392	4486	742	5228
19	35412	3109	570	3679
20	60421	3896	606	4502
21	32740	2141	380	2521
22	33369	2491	421	2853

**Total**	**2,435,343**	**143,185**	**22,827**	**164,296**

**Table 5:** HapMap SNPs using YRI HapMap Samples

	**Yoruba HapMap Samples**			
**Chromosome**	**Total number of SNPs**	**Total number of blocks**	**Total number Interblock SNPS**	**Total number of blocks + Interblock SNPs**

1	209439	17517	4169	21686
2	238828	19081	5688	24769
3	184337	15409	3635	19044
4	174670	14673	2754	17427
5	176975	14478	3063	17541
6	187787	14073	3127	17200
7	149764	12884	2451	15335
8	158800	13069	2465	15534
9	128582	11602	3185	14787
10	147710	12065	3778	15843
11	136474	11261	2793	14054
12	130298	11142	2383	13525
13	112162	8767	1470	10237
14	88022	7549	1240	8789
15	77885	7979	1657	9636
16	78364	8334	1810	10144
17	62720	6622	1754	8376
18	87027	7466	5294	12760
19	39729	4514	1037	5551
20	68828	6397	1344	7741
21	37450	3717	744	4461
22	36468	3945	790	4735

**Total**	**2,712,319**	**232,544**	**56,631**	**289,175**

We also altered the D' value used to define the blocks from 0.7 to 0.9 for Chromosome 1 in the HapMap CEU population to determine if block definition had a large impact on our results. Using a D' value of 0.7 results in 2,039 fewer "independent" SNPs on chromosome 1 which extrapolates to 44,000 fewer "independent" SNPs across the genome. Using a more stringent value of D' = 0.9 results in 2,906 more "independent" SNPs on chromosome 1 which extrapolates to 63,932 more "independent" SNPs across the genome. Although this may increase the range of total SNPs across the genome from 120,000 to 228,000 it does not alter the exponent of the p-value or substantially affect the thresholds (Table 3).

We also defined blocks using two additional block definitions: the Gabriel method and the 4-gamete rule. The Gabriel method creates blocks using stringent criteria of LD with a D' upper bound >0.98 and a lower bound >0.70[12]. This creates smaller blocks with fewer SNPs within a block. The 4-gamete rule of Wang, based on Hudson and Kaplan determines blocks based on presumed recombination[13,14]. Using pairwise sets of SNPs it determines the frequency of observing all 4 possible 2-SNP haplotypes. If all 4 haplotypes are observed, this method assumes recombination has occurred. Table 6 shows the results of different block definitions for Chromosome 1 for the CEU HapMap samples. The Gabriel method results in a similar number of blocks, but the number of SNPs per block is greatly reduced resulting in more SNPs outside of the block that are still in LD but do not meet the stringent criteria of a "block". The 4-gamete rule results in fewer blocks and more SNPs outside of blocks that represent potential recombination events. To limit the dependence on LD we believe the solid spine of LD is the best method to capture the underlying LD and biological dependence of SNPs, and therefore we base our thresholds on this method.

**Table 6:** Altering Block Definitions for Chromosome 1

	Total Number of Blocks	Total Number of Interblock SNPs	Total Number of SNPs and Blocks	Average Number of SNPs per block	Average D' per block
Solid Spine LD	10740	1815	12555	18.4	0.804

Gabriel	10115	38037	48152	15.7	0.805

4-Gamete Rule	18967	9084	28051	9.5	0.841

The method we detail is an extension to the original Bonferroni correction which is widely utilized; however, we have reduced the total number of SNPs to reflect the number of "independent SNPs" since independence is an assumption of the Bonferroni correction. Therefore, our thresholds are based on the original Bonferroni calculation of 1/Total # of SNPs, 0.05/Total # of SNPs and 0.001/Total # of SNPs where the number of SNPs that we use is now a better estimate of the number of independent tests being performed. Therefore, our proposed method allows a Bonferroni correction that has less violation of the assumption of independence.

We have empirically defined thresholds for genome wide association studies to control the family-wise error rate while accounting for the interdependence of SNPs in linkage disequilibrium. The use of actual data provides us an opportunity to unequivocally characterize the underlying linkage disequilibrium structure in these two populations. We considered the use of simulations as has been done for single chromosomes by assigning haplotypes based on frequencies from inferred haplotypes of founders for a set number of replicates [11]. But the reality is that simulation programs have thus far been unable to recreate the complexity of the underlying LD structure of the human genome. While we could use real 500 K genotype data and simulate unassociated traits, we would need to obtain many real 500 K GWAS data sets and then simulate many replicates of unassociated traits in each of them to adequately examine Type I error. Currently, this is a daunting task since the process just for obtaining the data from public databases is quite lengthy and the analysis time required to perform hundreds of GWAS analyses would be prohibitive.

By identifying the "independent" SNPs, we have significantly reduced the total number of SNPs to be used for Bonferroni correction in the set of SNP panels (Affymetrix and Illumina) and in HapMap. These "independent" SNPs provide us with a more accurate number of SNPs to include when adjusting for multiple testing using the Bonferroni correction. In addition, these p-values can assist in determining power for GWAS prior to genotyping so that only studies which can attain suggestive or significant association are pursued. We acknowledge that although we reduce the number of independent SNPS, the corresponding p-value cutoffs are still very low because we are analyzing more than 2 million SNPs without a specific biological hypothesis and stringency is still important. We need to balance identifying a true association while limiting Type 1 error.

We did evaluate the effects of the new thresholds on power using the Genetic Power Calculator to [15] determine the sample sizes we would need using a significance level based on all HapMap SNPs versus only the independent SNPs and blocks, as we recommend here. Table 7 provides different sample sizes using the 'LD adjusted' Bonferroni correction that we suggest here and the unadjusted Bonferroni correction in both CEU and YRI HapMap samples. Using the unadjusted Bonferroni correction would result in a necessary increase in sample size of 358–890 cases depending on the genotype relative risk and population. This increased burden of sample recruitment, collection and genotyping to adjust for "all" SNPs needs to be considered carefully, especially since many of the SNPs will be in strong LD and not contributing increased information.

**Table 7:** Examples of sample sizes required to have 80% power to attain significant association (family-wide error of 0.05) when using 'LD-adjusted' and unadjusted Bonferroni-corrected significance thresholds in CEU and YRI under different genetic models

P-value	Population	Genotype Relative Risk Aa/AA	Sample Size
3.04 × 10^-07^	CEU HapMap LD adjusted	1.4	5270 (-890)

2.08 × 10^-08^	CEU HapMap	1.4	6160

3.04 × 10^-07^	CEU HapMap LD adjusted	1.6	2550 (-431)

2.08 × 10^-08^	CEU HapMap	1.6	2981

1.73 × 10^-07^	YRI HapMap LD adjusted	1.4	5457 (-742)

1.85 × 10^-08^	YRI HapMap	1.4	6199

1.73 × 10^-07^	YRI HapMap LD adjusted	1.6	2641 (-358)

1.85 × 10^-08^	YRI HapMap	1.6	2999

Sample size is calculated with a high risk allele frequency of 10%, disease prevalence of 20%, and power of 0.80, with a difference in allele frequency between the causal marker and the genotyped marker of 10% (D' = 1.0). Sample size indicates the number of cases required (an equal number of controls is also required). The number in parentheses for sample size indicates the difference between the sample size required when using the LD adjusted Bonferroni correction versus using the unadjusted Bonferroni correction (which corrects for 2.4 million CEU HapMap SNPs and 2.7 million YRI HapMap SNPs.

## Conclusion

The emerging trend towards genome wide association studies and large scale SNP genotyping warrants universal thresholds of significance, similar to those established by Lander and Kruglyak for LOD score genetic linkage analyses [16]. The dilemma facing many researchers is which regions to follow-up with dense SNPs or sequencing? To date, the most utilized threshold has been the arbitrary value set by the Wellcome Trust Case Control Consortium of 5 × 10^-7 ^[17]. Interestingly, our Bonferroni LD-adjusted values are similar to these two thresholds (nominal p-value = 3.04 × 10^-7 ^for CEU), but we also provide thresholds for suggestive and highly significant association. We believe the *suggestive *association threshold should be used to identify SNPs for consideration in follow-up studies, and both the *significant *and *highly significant *associations should be considered regions more likely of association. Of course, these thresholds are only guidelines that account for the interdependency of SNPs and investigators should carefully consider any regions with strong candidate genes or biologic plausibility even if they do not meet these thresholds. We also agree with the NHGRI/NCI working group on Replication in Association Studies that *all *statistically significant regions should be replicated using additional populations with adequate sample size to confirm any GWAS finding [18]. These thresholds should assist in replicating regions of true association.

## Authors' contributions

PD and EMG participated in the design and analysis of the study, interpreted the data, and participated in writing and revising the manuscript. TNH analyzed the data, and participated in revising the manuscript. JEBW participated in the design and interpretation of the study and participated in revising the manuscript. All authors have read and approved the final manuscript.
